# A Chemiresistive Nanosensor Array for Rapid and Sensitive VOC-Based Detection and Differentiation of Prosthetic Joint Infection-Relevant Pathogens in Enriched Human Synovial Fluid

**DOI:** 10.3390/bios16030156

**Published:** 2026-03-12

**Authors:** Derese Getnet, Taejun Ko, Deyu Liu, Buyu Yeh, Jennifer Dootz, Venkatasivasai Sujith Sajja, Subramaniam Somasundaram, Mya Wilkes, Krista Toler, Robert Hopkins, Xiaonao Liu

**Affiliations:** 1 Tao Treasures LLC (d/b/a NanoBioFAB), Frederick, MD 21703, USA; derese.getnet@nanobiofab.com (D.G.); taejun.ko@nanobiofab.com (T.K.); deyu.liu@nanobiofab.com (D.L.); buyu.yeh@nanobiofab.com (B.Y.); jennifer.dootz@nanobiofab.com (J.D.); sujith.sajja@nanobiofab.com (V.S.S.); 2 Department of Diagnostics Research and Development, Zimmer Biomet, Warsaw, IN 46580, USA; subramaniam.somasundaram@zimmerbiomet.com (S.S.); mya.wilkes@zimmerbiomet.com (M.W.); krista.toler@zimmerbiomet.com (K.T.)

**Keywords:** biosensor, rapid detection, sensitive detection, chemiresistive nanosensor array, electronic nose, VOC profiling, machine learning, support vector machine, prosthetic joint infection, synovial fluid, point-of-care

## Abstract

Rapid and actionable pathogen identification remains a major unmet need in the diagnosis of prosthetic joint infection (PJI). Current diagnostic approaches either provide rapid host response information without pathogen specificity or identify pathogens with delays of days to weeks. Here, we report a chemiresistive nanosensor array combined with machine learning analysis for same-day, pathogen-specific detection based on volatile organic compound (VOC) profiling. A 19-channel nanosensor array was first validated in vitro against a panel of ESKAPEE pathogens, achieving 96% mean classification accuracy using a radial-basis-function support vector machine (SVM) classifier. Data-driven optimization yielded a reduced six-sensor array with high signal-to-noise performance. The optimized platform was evaluated using pooled, uninfected human synovial fluid enriched 1:1 with nutrient media and spiked with *Staphylococcus aureus*, *Staphylococcus epidermidis*, or *Pseudomonas aeruginosa* across a range of 1–10^6^ CFU/mL. All infected samples were detected within 9 h, with distinct VOC signatures enabling accurate pathogen differentiation. Time-to-detection (TTD) demonstrated a strong inverse correlation with initial bacterial concentration, supporting semi-quantitative estimation of bacterial load. Negative controls remained at baseline throughout testing. This chemiresistive VOC-based biosensor platform demonstrates the potential to deliver rapid, integrated detection, identification, and burden estimation of metabolically active PJI pathogens, highlighting its promise for future point-of-care diagnostic applications.

## 1. Introduction

Prosthetic joint infection (PJI) is among the most serious complications after arthroplasty. When evaluating a patient with a painful joint prosthesis, clinicians face a critical, time-sensitive decision: is the failure aseptic loosening or infection? In practice, a definitive diagnosis of PJI benefits from confirming both (1) elevated host response biomarkers and (2) the presence of a pathogen, ensuring infection is distinguished from aseptic failure with confidence.

Current diagnostic pathways, however, are fragmented and often compromise accuracy. Biomarkers of the host response, such as C-reactive protein (CRP) and alpha-defensin, offer speed and, in the case of alpha-defensin, strong specificity for infection [1,2]. However, while these markers provide powerful evidence of a host response consistent with PJI, they do not identify the causative organism or even directly indicate the active presence of a pathogen. Conversely, pathogen detection is traditionally performed via microbial culture, which remains essential for confirming infection and guiding targeted therapy, yet culture is slow and poorly aligned with urgent clinical decision-making [3]. The multi-day to multi-week waiting period for culture results creates a diagnostic void, often forcing surgeons to make high-stakes decisions, such as whether to perform a one-stage or two-stage revision, and which antibiotic to load in a cement spacer is based on incomplete information. Furthermore, culture-negative results are not uncommon in PJI, with reported rates ranging from 9% to as high as 42%, depending on whether preoperative aspirates or intraoperative tissue cultures are considered [4].

Molecular diagnostics seek to close this gap by bypassing culture. Targeted multiplex PCR assays (e.g., the BioFire Joint Infection Panel) can identify common PJI pathogens within hours and have demonstrated improved sensitivity for fastidious organisms [5,6]. These platforms are highly specific with low false-positive rates, but their coverage is confined to predefined targets and therefore may miss uncommon or unexpected pathogens. Next-generation sequencing (NGS) provides an unbiased alternative capable of detecting virtually any microbial DNA and has shown higher sensitivity in culture-negative cases, with typical turnaround times of ~48 h [7,8]. However, NGS remains susceptible to false positives from contamination, and both PCR and NGS detect nucleic acids from live and nonviable organisms alike, complicating clinical relevance [9,10]. Thus, despite important progress, current molecular approaches have not yet delivered a rapid, reliable, and clinically actionable real-time pathogen diagnosis in routine care.

These limitations motivate alternative approaches that can rapidly report the presence of an organism and identity while preferentially reflecting metabolic activity. Microbial volatile organic compounds (VOCs) are direct byproducts of metabolism and can encode rich, species-dependent signatures. Chemiresistive nanosensor arrays are attractive for VOC sensing because they enable continuous, low-power, real-time monitoring and have a clear path toward miniaturization.

In this feasibility study, we developed a chemiresistive nanosensor array coupled with machine learning to (i) rapidly detect the presence of key PJI-relevant pathogens in an enriched synovial fluid model, (ii) differentiate pathogen species based on VOC fingerprints, and (iii) provide a semi-quantitative estimation of bacterial load using a time-to-detection (TTD) metric. The overarching goal is to establish a foundation for future point-of-care, same-day, pathogen-specific testing in suspected PJI.

## 2. Materials and Methods

### 2.1. Bacterial Strains and Culture Conditions

The platform’s foundational algorithm was first established using a panel of seven clinically important (ESKAPEE) bacterial strains obtained from the American Type Culture Collection (ATCC, Manassas, VA, USA): *Enterococcus faecium* (BAA-2127, EF), *Staphylococcus aureus* (29213, SA), *Klebsiella pneumoniae* (27736, KP), *Acinetobacter baumannii* (BAA-1710, AB), *Pseudomonas aeruginosa* (27853, PA), *Escherichia coli* (11775, ES), and *Enterobacter cloacae* (BAA-3274, EC). In addition to the individual monomicrobial conditions, a 1:1 mixed culture of *S. aureus* (29213) and *P. aeruginosa* (27853) (PA-SA mixed) was also tested to simulate polymicrobial infection.

For each experiment, a single colony was inoculated into Luria–Bertani (LB) broth (Gibco™, Thermo Fisher Scientific, Waltham, MA, USA) and incubated overnight at 37 °C with shaking at 200 rpm. Cultures were then diluted and grown to an optical density corresponding to approximately 1 × 10^8^ CFU/mL. A 10 μL aliquot of each culture was spotted onto LB agar plates (EZ BioResearch, St. Louis, MO, USA) for VOC monitoring.

### 2.2. Chemiresistive Nanosensor Array and VOC Monitoring

VOC emissions from the agar plate cultures were monitored in real-time using a 19-channel chemiresistive sensor array (iNose^TM^, NanoBioFab (NBF), Frederick, MD, USA). The array was rationally designed based on a comprehensive literature-reported VOC associated with ESKAPEE organisms to ensure broad coverage of key chemical families, including alcohols (e.g., 2-butanol), ketones (e.g., 2-nonanone), sulfur compounds (e.g., dimethyl sulfide), aldehydes (e.g., acetaldehyde and 3-methylbutanal), and other metabolites crucial for differentiating bacterial species, which are representative VOCs reported across ESKAPEE pathogens (see Table 1 for full sensor list) [11,12,13].

**Figure 1:**
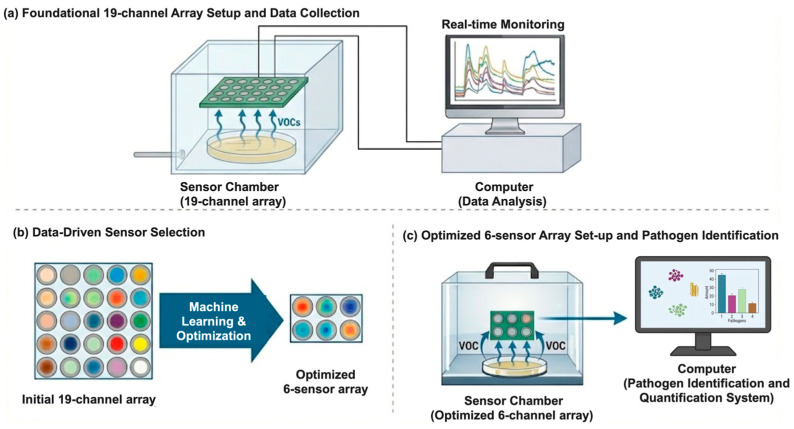
Schematic overview of the chemiresistive nanosensor array platform and experimental workflow for rapid pathogen detection. (**a**) Foundational setup for in vitro VOC profiling. A rationally designed 19-channel nanosensor array is placed within a controlled sensor chamber to continuously monitor the headspace VOC emissions from cultured bacterial plates. Sensor signals are recorded by a multichannel data acquisition system for real-time resistance monitoring and downstream analysis. (**b**) Data-driven sensor down-selection. Machine learning techniques, including principal component analysis (PCA), support vector machine (SVM) classification, and correlation matrices, were applied to the foundational dataset to eliminate redundant features, resulting in an optimized, high-performance 6-sensor array. (**c**) Application of the optimized 6-sensor array for clinical matrix testing. The miniaturized array monitors the headspace of enriched synovial fluid samples, capturing distinct VOC signatures to enable rapid, species-specific pathogen identification.

The iNose™ array utilizes a proprietary sensor platform that includes bespoke nanomaterial formulations that were developed, doped, and synthesized at NBF. Specifically, the sensing layer features a unique, highly uniform porous nanostructure (as confirmed via scanning electron microscopy, Supplementary Figure S1). This engineered porosity maximizes the surface-to-volume ratio, significantly improving the sensitivity for detecting trace microbial gas molecules. The array does not rely on commercially available off-the-shelf gas sensors; rather, the sensing elements are engineered via specific metal oxide compositions and functionalization strategies (protected under U.S. Patent Nos. US10780411B2, US20190360960A1, and US20240426806A1) to target broad classes of microbial VOCs. The schematic diagram in Figure 1 demonstrates the deployment of the 19-channel testing apparatus (including the controlled environmental chamber and the real-time multichannel data acquisition system). The 19 elements were used as a cross-reactive chemiresistive sensor array to capture multivariate VOC fingerprints. The array was placed over the inoculated plates to monitor the headspace for 48 h.

### 2.3. Sensor Down-Selection and Optimized Array

Following foundational development, we performed data-driven down-selection to optimize the array for pathogen detection in synovial fluid. Analysis of the foundational dataset revealed informational redundancy, where multiple sensors exhibited highly correlated responses to the same pathogen profiles. Sensor correlation matrices were applied to the in vitro dataset to identify a minimal, non-redundant subset of six sensors. The selection also prioritized sensors with the lowest power consumption, highest signal-to-noise ratios, and fastest response times—criteria critical for future point-of-care (POC) translation.

This foundational development resulted in a six-sensor array consisting of sensors S1, S3, S9, S10, S13, and S15. To facilitate scalable testing and advance toward POC miniaturization, these optimized sensor units were fabricated on miniaturized silicon chips (Figure 1b). We subsequently optimized the sensor formulation to enhance stability. For clarity in subsequent discussions, these optimized sensors were relabeled as follows: Sensor 1 = enhanced S1, Sensor 2 = enhanced S3, Sensor 3 = enhanced S9, Sensor 4 = enhanced S10, Sensor 5 = enhanced S13, and Sensor 6 = enhanced S15.

This systematic sensor down-selection process was a practical and necessary step in our pilot study. It served to demonstrate the diagnostic efficacy of the optimized six-sensor subset based on the foundational in vitro dataset before advancing to the highly complex synovial fluid application model, thereby providing critical guidance for future point-of-care hardware miniaturization.

### 2.4. Synovial Fluid Samples and Spiking

The optimized array was tested using pooled, de-identified, uninfected human synovial fluid (CD Laboratories, Towson, MD, USA). Remnant synovial fluid was previously de-identified in accordance with the institutional review board (IRB)-approved protocol (Western IRB-Copernicus Group [WCG] IRB). A total of 205 samples from knees (*n* = 190), hips (*n* = 11), and shoulders (*n* = 4) were pooled and used for testing. Samples were confirmed as uninfected based on clinical diagnostic testing. Evaluation of sample integrity included absorbance at 280 nm (between 0.342 and 1.18) and red blood cell count (RBC) ≤ 1,000,000/µL [14]. Additionally, biomarker tests showed white blood cell count (WBC) ≤ 3000/µL, C-reactive protein (CRP) ≤ 4.45 mg/L [15], α-defensin < 1, percentage polymorphonuclear leukocytes (%PMN) ≤ 70%, and negative culture results [16].

To ensure robust VOC signal generation, synovial fluid samples were enriched 1:1 with either LB (Gibco™, Thermo Fisher Scientific, Waltham, MA, USA) or Fastidious Broth (FB) (Remel™, Thermo Scientific™, Lenexa, KS, USA; Catalog No. R07664). The enriched samples were then spiked with PJI-relevant pathogens (*S. aureus*, *S. epidermidis*, and *P. aeruginosa*) at concentrations ranging from 1 to 10^6^ CFU/mL. The same ATCC reference strains described above were used for these experiments. Unspiked enriched fluid served as the negative control. Sample sizes for each condition are shown in Table 2.

For accurate and reproducible headspace monitoring of these liquid clinical matrices, the optimized 6-sensor array was integrated into a customized, 3D-printed multichannel testing fixture. This modular fixture securely positions each sensor unit directly above the individual synovial fluid sample wells, ensuring a consistent headspace volume and reliable VOC capture across all replicates.

### 2.5. Data Acquisition and Pre-Processing

For all experiments, the resistance value of each sensor was recorded every 10 s using a custom-built Multichannel Resistance Measurement System (NBF R-Scan v1.0; NanoBioFAB, Frederick, MD, USA). Raw data were subsequently down-sampled to 15 min intervals for analysis. The sensor response was normalized using the formula: Normalized Response = |ΔR/R_0_|, where R_0_ is the mean baseline resistance during the first hour of measurement, R is the instantaneous sensor resistance at a given time, and ΔR is the absolute change from baseline (R − R_0_).

Because the headspace of a multiplying bacterial culture contains a highly complex, dynamic mixture of hundreds of volatile metabolites encompassing both oxidizing and reducing compounds simultaneously, the array generates an aggregate net signal with mixed-direction responses (i.e., both resistance increases and decreases, as illustrated by the raw, signed response curves in Supplementary Figure S2). Therefore, the unsigned absolute response (|ΔR/R_0_|) was intentionally utilized in this study. The magnitude of signal perturbation from baseline provides the most robust and consistent mathematical feature for our multivariate machine learning classifier to differentiate overall pathogen presence from the sterile control, regardless of the net polarity of the complex mixture.

### 2.6. Detection Algorithm and Time-to-Detection (TTD)

VOC emissions from the synovial fluid headspace were monitored using the optimized 6-sensor array during incubation at 37 °C for up to 24 h. Key outputs included time-to-detection (TTD) and pattern recognition for pathogen detection and differentiation. TTD was defined as the elapsed time (in hours) from incubation start to the first positive call issued by the detection algorithm; samples that never crossed the decision boundary within the 24 h run were classified as negative.

### 2.7. Machine Learning and Statistical Analysis (Foundational In Vitro Dataset)

During the foundational development, principal component analysis (PCA) was used to visualize clustering among the nine classes (seven bacterial strains, one polymicrobial mixture, and one control). To further distinguish these classes, we trained a multiclass support vector machine (SVM) algorithm with a radial-basis-function (RBF) kernel on the 19-sensor feature set. (R 4.3.3, e1071). A sensor correlation matrix was used to remove redundant features, resulting in six sensors (S1, S3, S9, S10, S13, and S15). Performance was estimated via 10,000 Monte Carlo repetitions of stratified 75%/25% train–test splits: in each repetition, the model was fit on the training set and evaluated on the test set. Accuracy was computed in a one-vs-all scheme (proportion of correctly labeled samples for each class) and summarized across repetitions (and classes) to provide a stable estimate. Time-series box plots were generated for synovial fluid data to illustrate sensor dynamics across bacterial loads and controls.

## 3. Results

### 3.1. Foundational In Vitro Development

**Figure 2:**
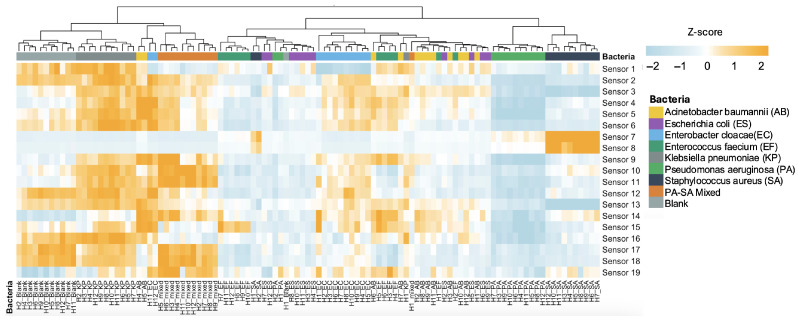
Heat map of 64 stable sensing features, extracted from 19 different nanomaterial-based sensors on the nanosensor array. Each row shows a specific bacterial group at a particular hour (the label ‘H1_SA’ denotes the sensor array readings at Hour 1 for *Staphylococcus aureus* (SA)), and each column displays the standardized readings from a different sensor. The color intensity reflects the standardized sensor value, ranging from cool blue to warm orange. Blue indicates a lower sensing value relative to other samples in that sensor, while orange indicates a higher sensing value. Hierarchical clustering shows samples of the same species cluster across time, indicating consistent sensor responses. Rows are ordered by similarity of z-scored profiles (Euclidean distance, Ward’s linkage); thus, IDs may not appear in time order.

The platform’s core ability to identify diverse pathogens was confirmed in vitro. The 19-sensor array captured distinct VOC fingerprints from all tested ESKAPEE pathogens, with unique signatures emerging within the first hour of monitoring. The heatmap in Figure 2 shows clear patterns that separate each microbial species over time. Blue indicates a lower sensing value relative to other samples in that sensor, while orange indicates a higher sensing value. Each pathogen sample has its own unique 19-sensor “signal pattern”. Samples with similar patterns are grouped together, and those from the same microbial species usually end up in the same group, much like sorting similar fingerprints into the same category. For example, KP exhibits the highest sensing values across most sensors, whereas PA shows the lowest.

**Figure 3:**
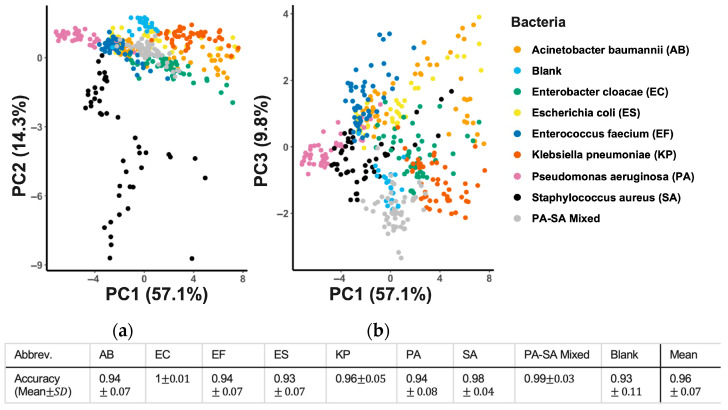
(**a**) Principal component analysis (PCA) plot of the sensing signals for differentiating ESKAPEE bacteria. Each point represents a sensor array readout. Each ESKAPEE bacterium, including a 1:1 PA-SA mixture, was individually cultured on LB agar plates and monitored continuously by the sensor array. (**b**) The table shows that the SVM classifier achieved an average accuracy of 0.96.

PCA confirmed this high degree of separation, with the first three principal components explaining 80% of the dataset’s variance and showing distinct, minimally overlapping clusters for each pathogen (Figure 3). Each point is a sample colored by class (AB (*Acinetobacter baumannii*), EC (*Enterobacter cloacae*), EF (*Enterococcus faecium*), ES (*Escherichia coli*), KP (*Klebsiella pneumoniae*), PA (*Pseudomonas aeruginosa*), SA (*Staphylococcus aureus*), Blank, and the SA–PA mixed set). The left panel shows PC1 vs. PC2; the right panel shows PC1 vs. PC3. Across both two-dimensional projections of the principal components, classes form recognizable clouds with limited overlap, indicating that a small number of orthogonal components capture most between-class variance. The SA cluster (black) lies largely distinct from other taxa. Gram-negative groups (AB, EC, KP, PA, and ES) occupy partially overlapping but ordered bands along PC1, with additional spreading on PC3 that helps tease apart KP and PA from AB/ES/EC. The SA–PA mixed group (light gray) falls between the SA and PA regions as expected. Blank samples (light blue) cluster tightly and away from most bacterial points, indicating low background variation and good separation from true positives.

The mean accuracy of the SVM classifier model across all runs was 96%. Class-wise accuracy was computed analogously within each class; the mixed-pathogen class reached 99.8% mean accuracy (Figure 2), indicating excellent separability. This foundational success established the platform’s high fidelity for the more challenging application in synovial fluid.

### 3.2. PJI Application Model: Pathogen Detection in Synovial Fluid

In the PJI application model, the platform successfully provided rapid and multi-dimensional answers to the three key questions.

#### 3.2.1. Nutrient Enrichment Is Required for VOC-Based Detection

**Figure 4:**
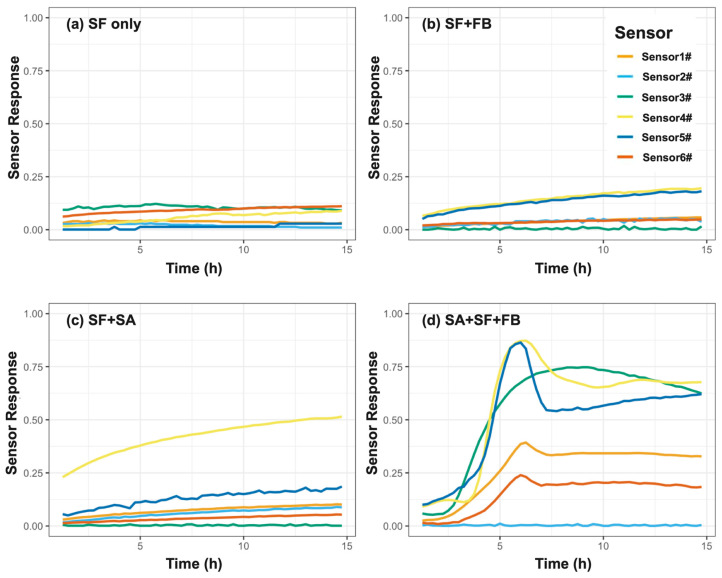
Nutrient enrichment is required for VOC-based pathogen detection. Real-time sensor responses from the 6-sensor chemiresistive array are shown under four experimental conditions: synovial fluid (SF) alone, SF with fastidious broth (SF+FB), SF spiked with *Staphylococcus aureus* (SF+SA), and SF with both *S. aureus* and fastidious broth (SA+SF+FB). The normalized response on the *Y*-axis is a dimensionless ratio calculated as |ΔR/R_0_| and expressed as a percentage. No significant signal was observed in control conditions (panels 1–3), indicating that neither the base matrix, enrichment medium, nor bacterial inoculation alone produced sufficient VOCs for detection. In contrast, robust and dynamic responses were observed in the enriched bacterial condition (panel 4), with multiple sensors (S3, S4, and S5) showing a sharp rise in signal between hours 4 and 8. This confirms that nutrient enrichment is essential for initiating metabolically active VOC signatures.

To determine whether nutrient enrichment was necessary for VOC-based detection, we tested four experimental conditions using synovial fluid (SF): (1) SF alone, (2) SF spiked with *Staphylococcus aureus* (SA), (3) SF supplemented with fastidious broth (FB), and (4) SF with both SA and FB (Figure 4). Conditions (1) through (3) served as negative controls. None of these control conditions generated significant VOC signals. Sensor responses remained near baseline throughout, indicating that neither the neat synovial fluid, the enrichment medium alone, nor bacterial inoculation in SF was sufficient to elicit detectable metabolic activity. In contrast, when SA was incubated in enriched synovial fluid (SA+SF+FB), a rapid and dynamic VOC response emerged. Multiple sensors (particularly Sensor 4 and Sensor 5) showed a pronounced signal increase, peaking between 6 and 8 h before stabilizing. This confirmed that nutrient enrichment is a prerequisite for enabling bacterial metabolic activation and subsequent VOC-based detection.

#### 3.2.2. Rapid and Sensitive Detection Across Inocula

**Figure 5:**
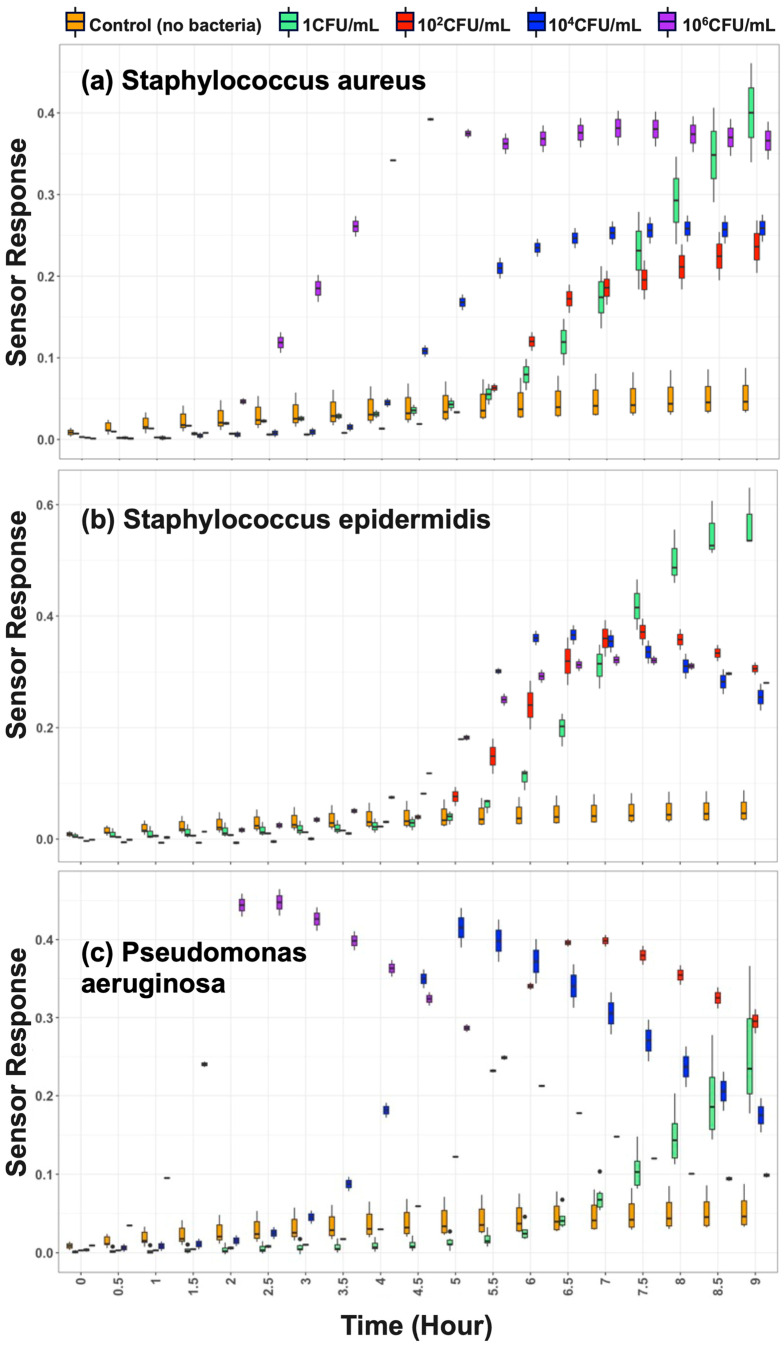
Time-to-detection across a range of bacterial inocula for three PJI-relevant pathogens. Normalized VOC signal responses over time are shown for a representative sensor under varying inoculum concentrations (1 to 10^6^ CFU/mL) for (**a**) *Staphylococcus aureus* (SA), (**b**) *Staphylococcus epidermidis* (SE), and (**c**) *Pseudomonas aeruginosa* (PA). Boxplots represent sensor response distributions across replicates. In all cases, detection time decreased as the inoculum increased, demonstrating a clear inverse correlation between bacterial load and time-to-detection. For example, in (**a**), SA at 10^6^ CFU/mL is detected at 2.25 h, while at 1 CFU/mL, it is detected at 6.5 h. Detection thresholds were defined using both fixed shift (ΔR/R_0_ > threshold sustained > 1 h) and statistical envelope criteria (see Section 2).

Using this enrichment-based workflow, the platform reliably detected bacteria across a wide range of inocula (1 to 10^6^ CFU/mL). As shown in Figure 5, binary “positive” or “negative” results were generated in under 9 h, while negative controls remained at baseline throughout the monitoring period, confirming specificity.

#### 3.2.3. Pathogen Differentiation

Beyond presence/absence detection, the platform differentiated the specific spiked pathogen species. As shown in Figure 5, pairwise analyses using six-sensor panels showed clear separation among *S. aureus*, *S. epidermidis*, and *P. aeruginosa*. Notably, even two sensors (Sensor 1 and Sensor 6) provided discriminative patterns sufficient for clustering at 10^4^ CFU/mL.

**Figure 6:**
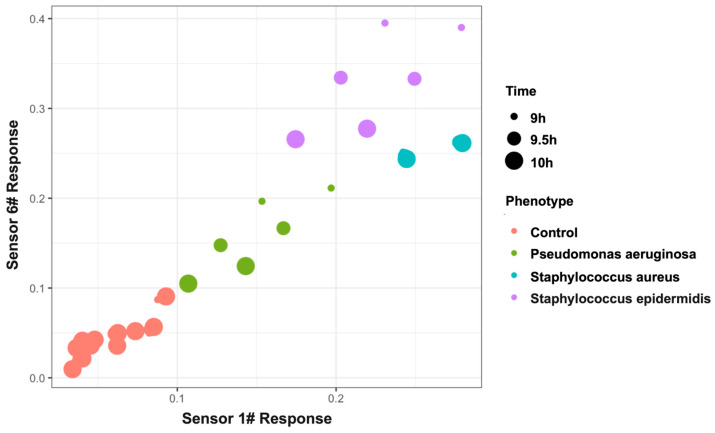
Scatter plot demonstrating the spatial clustering and differentiation of major PJI-relevant pathogens (*S. aureus*, *S. epidermidis*, and *P. aeruginosa*) at a moderate clinical bacterial load of 10^4 CFU/mL. The X and Y axes represent the normalized response values (calculated as |ΔR/R_0_|) of two highly discriminative sensors (Sensor 1 and Sensor 6) derived from the optimized array. This two-dimensional projection visually illustrates that distinct spatial clustering effectively separates the pathogens based on their unique combinatorial VOC fingerprints.

Because the sample sizes per specific concentration node (ranging from 1 to 10^6^ CFU/mL) are inherently limited in this initial proof-of-concept phase, calculating a formal statistical classification accuracy metric for each discrete concentration was deferred to avoid mathematical overfitting. Nevertheless, to demonstrate that varying initial bacterial burdens do not abolish the platform’s underlying combinatorial differentiation capability, spatial clustering was evaluated. As shown in Figure 6, the platform successfully maintains distinct pathogen differentiation even at a moderate clinical load of 10^4^ CFU/mL.

#### 3.2.4. Semi-Quantitative Burden Estimation via TTD

**Figure 7:**
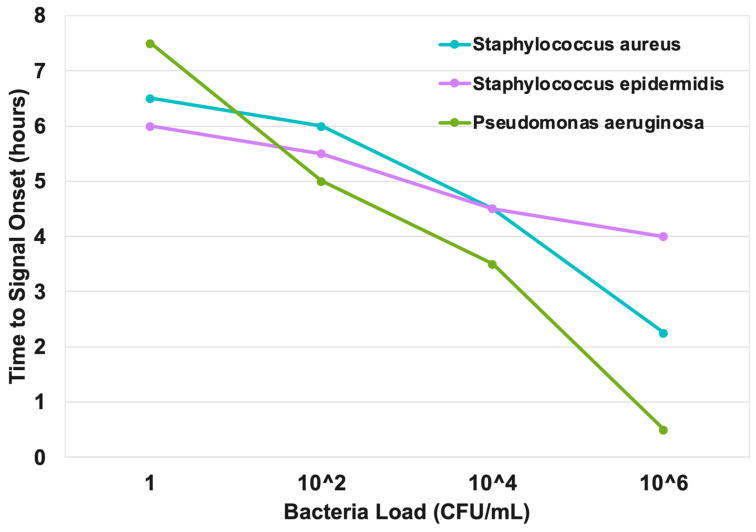
Time to signal onset for *S. aureus*, *S. epidermidis*, and *P. aeruginosa* is plotted against increasing bacterial loads. As expected, the higher the bacterial load, the faster the signal onset. *P. aeruginosa* demonstrated the fastest signal onset at higher concentrations.

The platform enabled semi-quantitative estimation of the microbial burden by correlating time-to-detection (TTD) with the initial bacterial load. The time-resolved data show a clear dose-dependent response for all three pathogens (Figure 5). For instance, in the case of *S. aureus*, a high load of 10^6^ CFU/mL was detected at ~2 h, whereas 1 CFU/mL required ~6.5 h (Figure 5a). Similar patterns were observed for *S. epidermidis* (~4 h at 10^6^ vs. ~6 h at 1 CFU/mL) and *P. aeruginosa* (~0.5 h at 10^6^ vs. ~7.5 h at 1 CFU/mL) (Figure 5b,c). This strong inverse correlation between bacterial load and TTD is summarized in Figure 7.

## 4. Discussion

This study demonstrates the feasibility of a chemiresistive nanosensor array platform to deliver same-day, actionable intelligence for PJI diagnosis within a controlled laboratory model. By addressing three fundamental diagnostic questions—(i) is a metabolically active organism present, (ii) if the organism is present, what is it, and (iii) if the organism is present, what is the bacterial load—this study addresses the unmet clinical diagnostic need for rapid detection and monitoring of bacteria in PJI diagnosis. We believe our approach in this proof-of-concept study for addressing these questions directly targets the most pressing unmet needs in current practice. Unlike existing methods that provide fragmented information, this platform integrates these insights into a unified report within hours.

The potential clinical value of such rapid, integrated intelligence is substantial. Early identification of the pathogen, such as distinguishing Gram-negative from Gram-positive organisms, could significantly influence surgical planning (e.g., one-stage or two-stage revisions) and targeted empirical antibiotic selection [17]. This capability could also enable more informed decisions regarding antibiotic-loaded cement spacers during two-stage revisions, a process often guided by incomplete preoperative data [18,19]. Similarly, the ability to estimate bacterial burden introduces a novel dimension to preoperative assessment. A high bacterial load might justify the use of more aggressive or specialized lavage solutions (e.g., antiseptic or citrate-based solutions) or influence the choice between one-stage and two-stage exchange arthroplasty [17,20,21].

Our diagnostic workflow incorporates a simple enrichment step to ensure robust metabolic activity for VOC analysis. While direct detection from neat synovial fluid was not feasible in this study, the enrichment-based approach enabled reliable pathogen detection within 9 h, which is substantially faster than conventional culture methods that typically require several days for results [22,23]. It is important to note that while this 9 h detection window represents a significant improvement over traditional agar plate cultures, modern automated blood culture systems (e.g., BACTEC) can also flag microbial growth within comparable timeframes for high-burden samples. However, the primary clinical advantage of our proposed VOC-based platform extends beyond mere speed; it lies in its integrated specificity. Unlike automated cultures that only flag the generic presence of growth and require subsequent days for sub-culturing and definitive organism identification, our platform provides potential same-day, pathogen-specific differentiation directly from the initial headspace profile. Furthermore, the finding that a reduced six-sensor array could perform this complex task underscores the platform’s strong potential for future point-of-care (POC) miniaturization and cost-effective device development.

Moreover, our finding that a higher bacterial load correlates with a faster time-to-detection (TTD) provides a valuable semi-quantitative dimension to the diagnostic result. This inverse relationship is consistent with prior observations from conventional blood culture systems, where higher inoculation amounts typically yield faster time-to-positivity (TTP) [24,25,26]. Although well recognized in the laboratory, such information has rarely been incorporated into clinical decision-making. Notably, in bloodstream infections, a shorter TTP is a robust surrogate for high-grade bacteremia and has been linked to increased sepsis severity and mortality. While less studied in PJI, it is clinically plausible that a similar principle applies, with higher initial microbial burden reflecting a more aggressive infection. Thus, our platform’s ability to provide rapid TTD on the same day as aspiration may serve not only to confirm infection but also as an early indicator of burden severity. Prospective clinical studies will be needed to validate the prognostic value of TTD in PJI management.

From a biosensor perspective, this platform differs fundamentally from traditional electronic-nose and GC-based VOC systems by employing a rationally designed chemiresistive nanosensor array optimized for microbial metabolic signatures rather than bulk chemical separation. Unlike GC–MS or mass spectrometry approaches, the chemiresistive array enables continuous, low-power, real-time monitoring. Furthermore, it is essential to contextualize the sensor array’s performance within its intended operational biological environment. In our experimental configuration, the ~100% relative humidity (RH) headspace at 37 °C effectively functioned as a highly consistent, non-variable background matrix corresponding to standard clinical incubation conditions, rather than a fluctuating experimental variable. While competitive water adsorption is a known challenge that can suppress sensitivity in traditional chemiresistive systems, our proprietary nanomaterials were specifically optimized to retain high chemical activity in this saturated environment. This retained sensitivity is conclusively demonstrated by the array’s robust ability to detect multiple PJI-relevant pathogens down to extremely low trace burdens of 1 CFU/mL directly within the high-humidity synovial fluid headspace (Figure 4 and Figure 5). Additionally, continuous monitoring demonstrated exceptional baseline stability in this environment, with maximum baseline drift remaining strictly below 5% over a prolonged 15 h period (Supplementary Figure S3).

Despite these promising feasibility results, several technical challenges and practical limitations must be explicitly addressed before this platform can transition into routine clinical care. First, it is crucial to clarify the sample sizes utilized across different phases of this study. While our foundational in vitro machine learning model achieved 96% classification accuracy using a comprehensive dataset validated with 10,000 Monte Carlo repetitions (Section 3.1), the sample sizes in the human synovial fluid application phase were inherently limited and imbalanced (ranging from *n* = 2 to *n* = 11 per condition, Table 2). Consequently, the synovial fluid findings serve strictly as a preliminary proof-of-concept utilized primarily to demonstrate signal initiation feasibility and map TTD, rather than to train a standalone generalized clinical classifier. Second, this proof-of-concept study was conducted using pooled uninfected synovial fluid seeded with a limited set of standard laboratory bacterial isolates, and it relied on a brief nutrient enrichment step to standardize VOC production. Real-world clinical samples are highly heterogeneous, may include fastidious organisms, and can be profoundly affected by prior patient antibiotic exposure. Third, translating this combinatorial fingerprinting approach from a controlled lab incubator to unsealed real-world clinical environments introduces the risk of unpredictable environmental VOC interferences (e.g., ambient clinical odors and sterilizing agents). Extensive future multi-center validation with statistically meaningful clinical cohorts, including both culture-positive and culture-negative suspected PJI cases, alongside advanced algorithmic background compensation, will be essential to assess diagnostic accuracy across the full biological diversity of real-world infections and confirm the generalizability of this approach.

## 5. Conclusions

This novel chemiresistive nanosensor platform demonstrates strong potential as a disruptive diagnostic approach for microbial detection and identification in the setting of PJI. This laboratory model provides rapid, specific, and semi-quantitative data on the presence, identity, and load of metabolically active microorganisms, which can address key gaps in current diagnostic workflows. If validated in clinical studies, this technology could transform PJI management by replacing a multi-day diagnostic process with same-day actionable intelligence, enabling more timely, targeted, and personalized treatment strategies.

## Figures and Tables

**Table 1:** Rational design of the 19-sensor array based on preferential cross-sensitivity (VOC classes of ESKAPEE pathogens).

Sensor Subset	# of Sensors	Primary Target VOC Chemical Classes	Relevance to Microbial Metabolism
S1, S6	2	Nitrogenous Compounds (e.g., indole and pyrazines)	Derived from tryptophan and other amino acid degradation, highly specific markers for certain enteric bacteria.
S2, S3, S4, S5, S19	5	Alcohols and Ketones (e.g., 2-pentanol, 2-butanone, and isoprene)	Products of amino acid and carbohydrate fermentation pathways, common across many bacterial species.
S7, S8, S9, S10, S11	5	Sulfur Compounds (e.g., dimethyl sulfide and methyl mercaptan)	Byproducts of methionine and cysteine metabolism; characteristic strong foul odors (e.g., in *P. aeruginosa*).
S12, S13, S16, S17	4	Aldehydes and Esters (e.g., acetaldehyde and amyl isovalerate)	Result from lipid metabolism and fatty acid degradation; contribute to species-specific “fruity” or “sharp” aroma profiles.
S14, S15, S18	3	Hydrocarbons and Organic Acids (e.g., tridecane and acetic acid)	General metabolic byproducts related to cell membrane components and the citric acid cycle.
Total	19	Broad-Spectrum VOC Coverage	Designed to capture diverse metabolic activities for robust pathogen differentiation.

For the PJI application model, the optimized subset was relabeled for readability as Sensors 1–6 in a fixed order (Sensor 1 = S1; Sensor 2 = S3; Sensor 3 = S9; Sensor 4 = S10; Sensor 5 = S13; Sensor 6 = S15). The assigned VOC classes represent preferential cross-sensitivity profiles of the rationally designed nanomaterials rather than absolute lock-and-key selectivity. The diagnostic capability of the array inherently relies on ensemble combinatorial fingerprinting and multivariate pattern recognition of complex gas mixtures.

**Table 2:** Summary of sample size per condition.

Species	Concentration	Sample Size
*P. aeruginosa*	1	6
*P. aeruginosa*	10^2^	2
*P. aeruginosa*	10^4^	2
*P. aeruginosa*	10^6^	2
*S. aureus*	1	3
*S. aureus*	10^2^	2
*S. aureus*	10^4^	2
*S. epidermidis*	1	3
*S. epidermidis*	10^2^	2
*S. epidermidis*	10^4^	2
*S. epidermidis*	10^6^	2
CONTROL	0	11

## Data Availability

All raw data are available on request.

## Supplementary Materials

The following supporting information can be downloaded at: https://www.mdpi.com/article/10.3390/bios16030156/s1.
