# Supplementary material for: A Chemiresistive Nanosensor Array for Rapid and Sensitive VOC-Based Detection and Differentiation of Prosthetic Joint Infection-Relevant Pathogens in Enriched Human Synovial Fluid
Source: Biosensors (Basel). 2026 Mar 12;16(3):156. doi: 10.3390/bios16030156 (PMC13023655; doi:10.3390/bios16030156)
Supplement: Supplementary file 1 [file biosensors-16-00156-s001.zip › biosensors-4154156-supplementary.pdf]

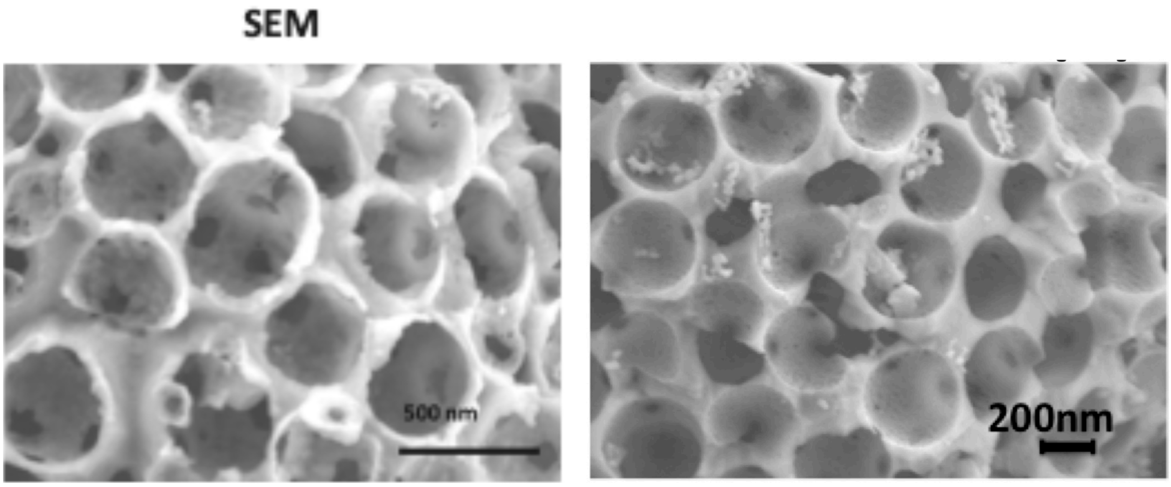

Figure S1: Sensing layer of nanosensor with unique, highly uniform porous nanostructure under Scanning electron microscopy (SEM)

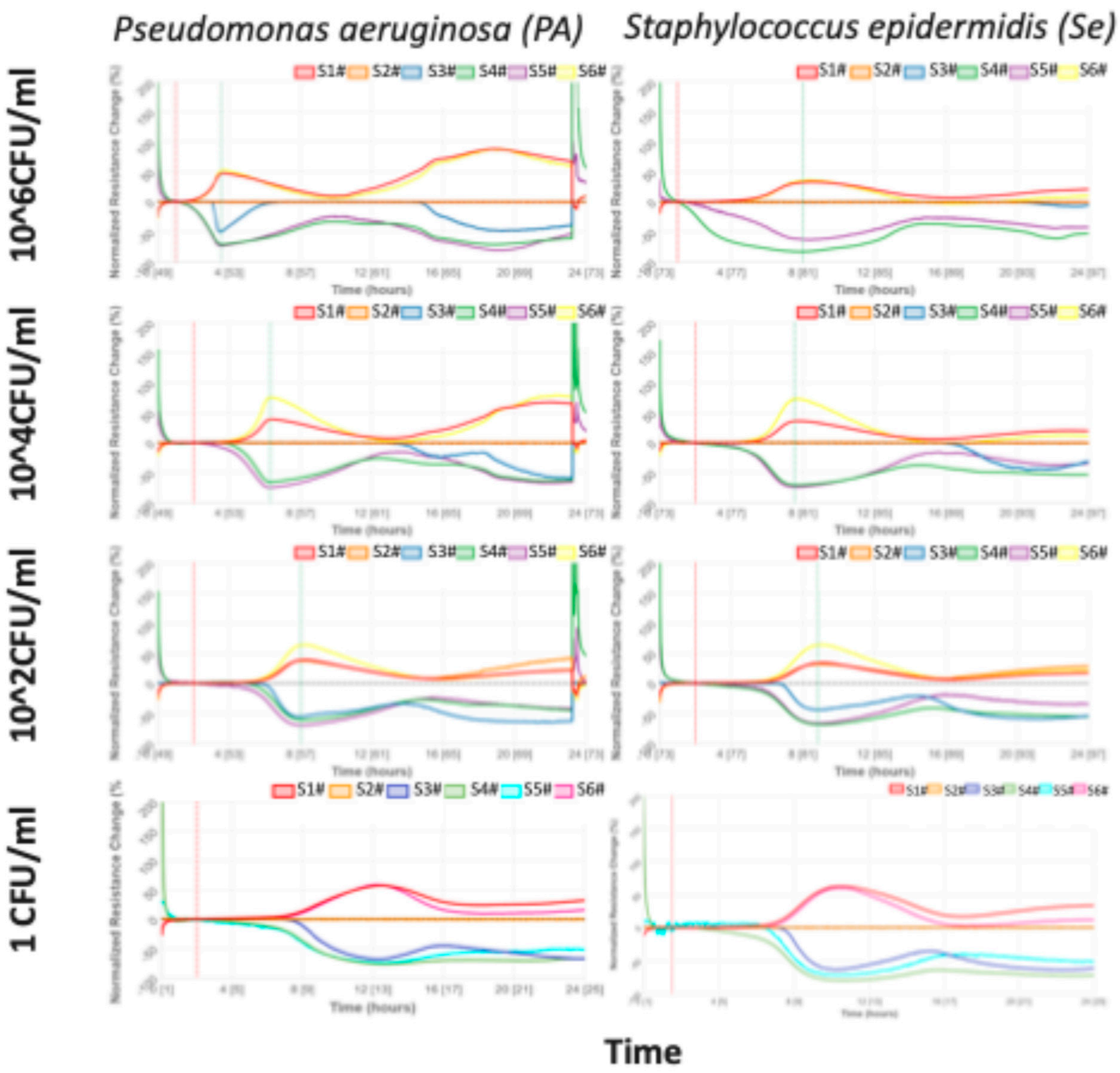

Figure S2. Raw, signed resistance response curves of the 6-channel sensor array exposed to a representative pathogen sample. The mixed-directionality (both resistance increases and decreases) reflects the highly complex nature of the bacterial headspace containing both oxidizing and reducing volatile metabolites.

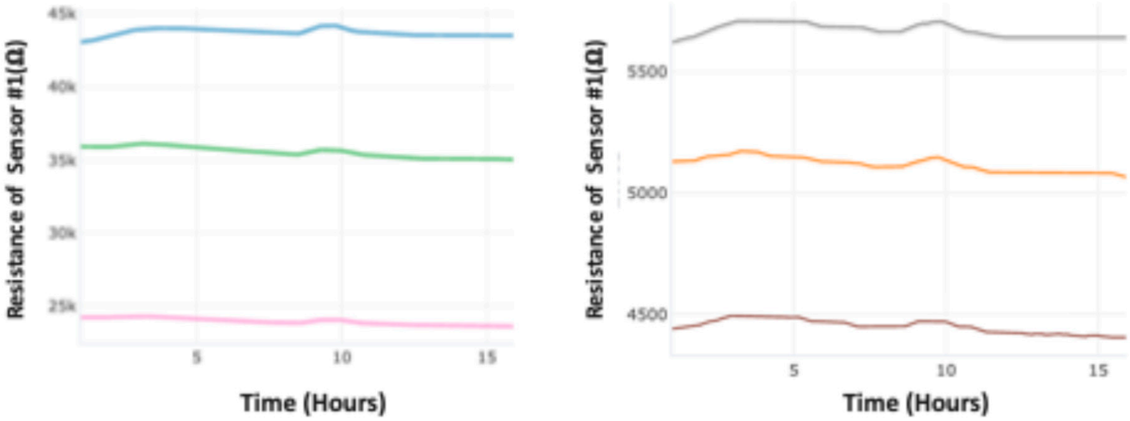

Figure S3. Continuous raw resistance monitoring for representative sensors (e.g., LW60 and LW65) over a prolonged 15-hour period in the sterile negative control group (unspiked Fastidious Broth). The maximum baseline drift remained strictly below 5%, demonstrating exceptional stability in the ~100% RH high-humidity testing environment.
